# Serum Hepcidin Levels in Childhood-Onset Ischemic Stroke: A Case-Control Study

**DOI:** 10.1097/MD.0000000000002921

**Published:** 2016-03-07

**Authors:** Seham F. Azab, Nagwa E. Akeel, Mohamed A. Abdalhady, Ahmed A. Elhewala, Al Shymaa A. Ali, Ezzat K. Amin, Dina T. Sarhan, Mohamed A.A. Almalky, Eman M. Elhindawy, Mohamed M.A. Salam, Attia A. Soliman, Sawsan H. Abdellatif, Sanaa M. Ismail, Nahla A. Elsamad, Mustafa I.A. Hashem, Khalid A. Aziz, Osama M.A. Elazouni, Manal S. Arafat

**Affiliations:** From the Faculty of Medicine, Zagazig University, Al Sharqia Governorate (SFA, NEA, MAA, AAE, ASAA, EKA, DTS, MAAA, EME, MMAS, AAS, SHA, SMI, NAE, MIAH, KAA, OMAE); and Faculty of Medicine, Mansoura University Student hospital, Dakahlia Governorate,Egypt (MSA).

## Abstract

Recently, hepcidin, an antimicrobial-like peptide hormone, has evolved as the master regulator of iron homeostasis. Despite the growing evidence of iron imbalance in childhood-onset ischemic stroke, serum hepcidin level in those patients has not yet been researched.

In this study, we aimed to estimate serum (hepcidin) level in acute ischemic stroke (AIS) patients and to investigate whether subcutaneous enoxaparin sodium, which is a low-molecular-weight heparin (LMWH) derivative, could modulate serum hepcidin level in those patients.

This was a case–control study included 60 (AIS) cases, and 100 healthy children with comparable age and gender as control group. For all subjects’ serum hepcidin, interleukin-6 (IL-6), and soluble transferrin receptor [sTfR]) levels were assessed by (enzyme-linked immunosorbent assay [ELISA] method). Iron parameters including (serum iron, ferritin, transferrin, and total iron binding capacity [TIBC]) were also measured. The patients were subdivided according to treatment with an LMWH derivative into 2 groups and serum hepcidin levels were assessed initially and 1 week after stroke onset for all cases.

We found that AIS cases had higher serum iron, ferritin, and IL6 levels compared to the control group (all *P* < 0.01). Serum hepcidin was significantly higher in AIS cases (median, 36[15–73]ng/mL) compared to the control group (median, 24[10–41]ng/mL; *P* < 0.01). On the 1st day of AIS diagnosis, serum hepcidin levels were similar in both stroke subgroups (*P* > 0.05). However, on the 7th day of diagnosis serum hepcidin level decreased significantly in AIS cases treated with LMWH (group 1) (median, 36 vs 21 ng/mL; *P* < 0.01, respectively). Meanwhile, no significant change was observed in serum hepcidin level in AIS cases not treated with LMWH (group 2) (*P* > 0.05). Serum hepcidin showed significant positive correlations with serum iron, transferrin saturation, ferritin, and IL6 (*r* = 0.375, *P* < 0.05; *r* = 0.453, *P* < 0.05; *r* = 0.687, *P* < 0.01; *r* = 0.515, *P* < 0.01; respectively).

Our data brought a novel observation of elevated serum hepcidin level in pediatric AIS patients and pointed out that treatment with LMWH could modulate hepcidin level in those patients.

## INTRODUCTION

Cerebro-vascular diseases are one of the top 10 causes of mortality and morbidity in children.^1^ Childhood-onset ischemic stroke is characterized by finding of arterial-distribution ischemia in a child age 29 days to 18 years. Patients typically present with sudden onset neurological deficits, but are often not diagnosed until over 24 hours thereafter.^2^ Many of these children are left with permanent neurologic deficits and epilepsy, and pediatric stroke incurs a high cost to families and societies.^3^

Iron, the most abundant trace element in the brain, is considered to be important for normal neurodevelopment. It is also believed to play a critical role in neuronal injury caused by oxidative stress in ischemia, although the exact mechanism is not fully understood.^4^

Hepcidin-25, a 25-amino acid peptide hormone produced in the liver, is a central regulator of systemic iron metabolism.^5,6^ Hepcidin downregulates duodenal iron absorption and macrophage iron release by modulating cellular iron export via ferroportin (FPN1).^7^At the molecular level, hepcidin binds to the sole known cellular iron efflux channel, FPN1, and induces its internalization and lysosome degradation by mechanisms similar to those that inactivate other more conventional membrane receptors.^7^ Disruption in FPN1 protein expression and the associated decline in the neuronal efflux of iron might ultimately lead to increased brain iron levels.^8^

Despite the growing evidence of iron imbalance in childhood-onset ischemic stroke, serum hepcidin level in those patients has not yet been researched.

In this study, we aimed to estimate serum (hepcidin) level in acute ischemic stroke (AIS) patients and to investigate whether subcutaneous enoxaparin sodium, which is a low-molecular-weight heparin (LMWH) derivative, could modulate hepcidin level in those patients.

## METHODS

This was a case–control study performed in Zagazig University Children Hospital ICU and Outpatient Clinics in the same Hospital from October 2012 to April 2015.

### Cases

Sixty AIS patients (45 males and 15 females) were included in our study. All had previously been generally healthy, their age ranged from 1 to 15 year (median 8 years). AIS was defined as a focal neurologic deficit of acute onset (e.g., aphasia, hemiparesis, hemi sensory loss, change in vision, or loss of balance) and a computed tomography scan or magnetic resonance image (MRI) scan of the brain showing a lesion characteristic of a focal arterial infarct in a vascular territory consistent with the neurologic presentation.^9^ Patients were enrolled if a diagnosis of definite stroke had been made by a neuroradiologist based on classical neuroradiological features consistent with a focal neurologic deficit of acute onset.^9^

### Exclusion Criteria

We excluded cases with hemorrhagic stroke, head trauma, CNS infection, congenital or acquired heart disease, malignancy, renal or hepatic dysfunction, cerebrovascular malformation, sickle cell anemia, recent surgery, chronic inflammatory disease or chronic infection, iron deficiency anemia, or regular iron supplement therapy within the most recent 3 months.

### Control Group

One hundred healthy children, of comparable age and gender, who attended Pediatric Department for preoperative evaluation for elective surgery, were enrolled.

As ferritin, an acute phase reactant may be elevated during comorbid infection(s), we excluded any child presented with any evidence of infections or inflammations or history of iron therapy in the previous 3 months.^10^ All patients and controls included were subjected to proper history taking, thorough clinical and detailed neurological examination.

Brain MRIs, magnetic resonance angiography, and magnetic resonance venography were performed to all stroke patients.

Laboratory investigations were done for all studied children and included: urine analysis, stool analysis, complete blood count (CBC) including blood indices, ESR, and C-reactive protein (CRP), urine culture and sensitivity test, liver, and kidney functions tests.

We divided our AIS cases into 2 groups:

1. Group 1(n = 30): AIS patients who received subcutaneous enoxaparin sodium, which is a LMWH derivative (Enoxaparinum natricum, Clexane; Sanofi–Aventis, Paris, France). Enoxaparin was administered at a dose of 1 mg/kg subcutaneously every 12 hours daily from the 2nd day onward during the patients’ hospital stay.^11^
2. Group 2 (n = 30): AIS patients who did not receive (LMWH).

For ethical consideration, only AIS patients whose baseline laboratory investigations, echocardiography or neuroimaging revealed contraindication to LMWH administration, were included in group 2 (Table 1).

**TABLE 1:**
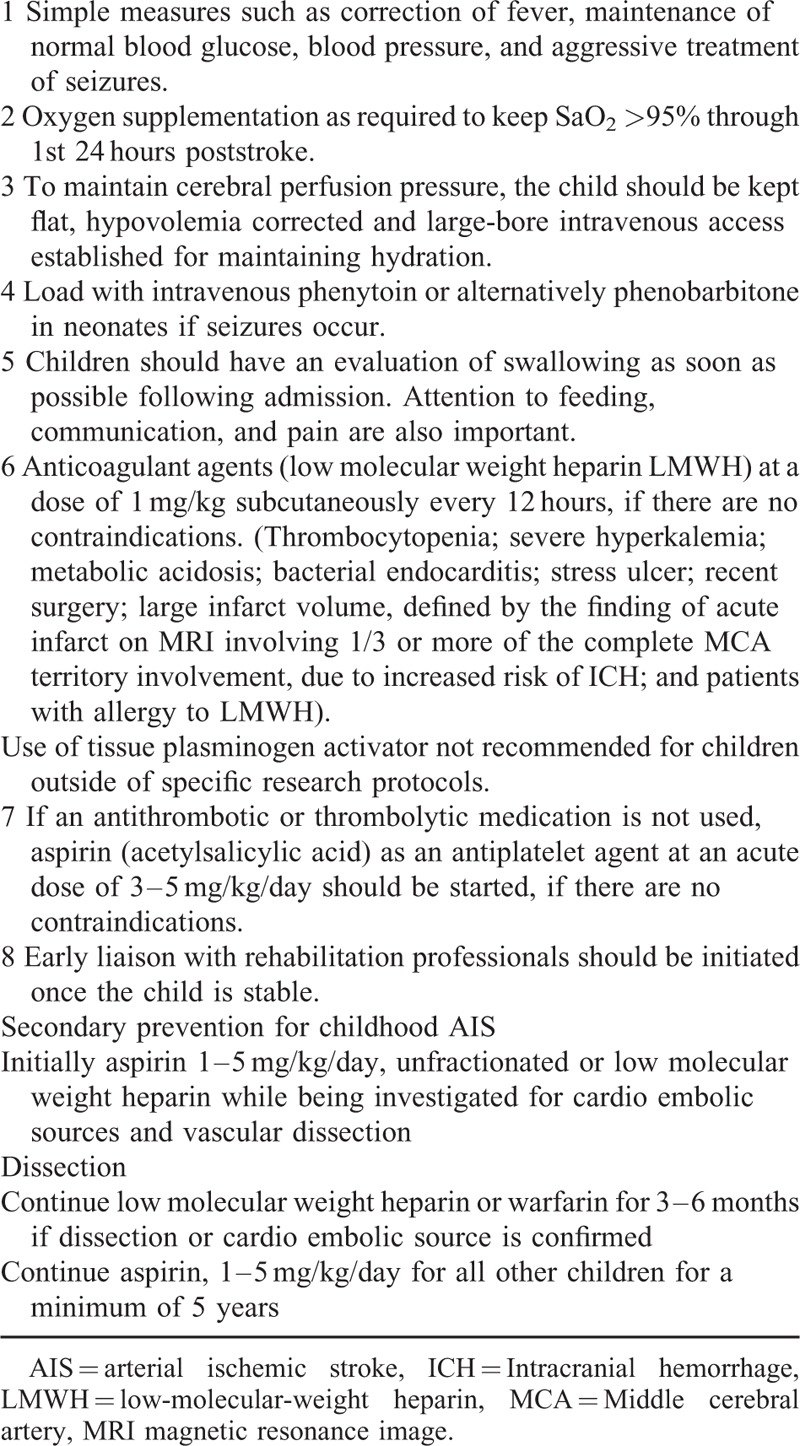
AIS Treatment Protocols in Our Hospital Included

The blood sample was obtained from all patients immediately after ICU admission which occurred within 8 hours after stroke onset (mean ± SD time after stroke onset, 5.4 ± 1.6 hours). Blood samples were drawn using metal-free and stainless steel needles into appropriately coated tubes (Becton Dickinson Laboratories, Franklin Lakes, NJ) for measurement of serum levels of iron. The tubes were centrifuged at 2000×g for 10 minutes and the samples were tested using Inductively Coupled Plasma mass-spectrometry (ICP-MS, Perkin Emler Optima 4300, DV, USA).^12^ Serum ferritin was analyzed by an immuno-assay (Elecsys, BoehringerManneim, France).

### Iron Parameters

Serum was analyzed for ferritin, iron, total iron binding capacity (TIBC), transferrin saturation (Iron/TIBC), and C-reactive protein (CRP) levels. The soluble transferrin receptor (sTfR) level was measured by a competitive enzyme-linked immunosorbent assay (ELISA, C-ELISA) (R&D Systems, Inc., Minneapolis, MN).

### Serum Hepcidin-25 Measurement

Peripheral venous blood samples were obtained from each stroke patient 2 times: at the time of diagnosis (1st day) and 1 week after diagnosis (7th day). Fasting control blood samples were collected from healthy subjects only once. Serum hepcidin-25 levels were measured by a C-ELISA using a commercial kit from Peninsula Laboratories (Bachem, Torrance, CA) as described previously.^13^ Patients’ samples were assayed in duplicate. The results from the C-ELISA were compared with those of the standard curves developed from calibrators run simultaneously with study samples.

### Measurement of Serum Interleukin-6 (IL-6) Levels

The concentrations of IL6 in serum were estimated using a double antibody sandwich ELISA (kit provided by Biosource Europe S.A., Belgium) according to the manufacturer's instructions by using standard curve.

### Ethics

Informed parental consent was obtained to be eligible for enrollment into the study. The study was done according to the rules of the Local Ethics Committee of Faculty of Medicine, Zagazig University, Egypt. Our institutional review committee of ethical research approved the study.

### Statistical Analysis

SPSS version 20 and EPI-info Version 6.04 were used for data analysis. The data are expressed as the mean ± SD or median (min–max) where appropriate. Test selection was based on evaluating the variables for normal distribution using the Shapiro–Wilk test. If the variables had a normal distribution, Student's *t*-test was used. If the variable did not have a normal distribution, the analysis was done using the Mann–Whitney *U*-test. Categorical data were evaluated by Pearson's Chi-square test. The Wilcoxon signed-rank test was used to verify the difference of hepcidin levels between the times of stroke diagnosis (1st day) and 1 week after stroke diagnosis (7th day). Statistical correlations were calculated by Spearman correlation test. *P* < 0.05 was considered significant.

## RESULTS

There was no significant difference between AIS cases and control group as regards age, gender, or BMI (*P* > 0.05), respectively (Table 2). Serum iron, transferrin saturation, and ferritin were significantly higher in AIS cases compared to the control group (all *P* < 0.01). TIBC was significantly lower in stroke cases compared to the controls (*P* < 0.01). Meanwhile, there were no significant differences between studied groups as regards serum transferrin or sTfR; *P* > 0.05 (Table 2). Interestingly, serum hepcidin was significantly higher in AIS cases (median, 36[15–73] ng/mL) compared to the control group (median, 24[10–41] ng/mL; *P* < 0.01) (Table 2). As expected, stroke cases had increased levels of both serum IL6 and CRP compared to the control group; *P* < 0.01 (Table 2).

**TABLE 2:**
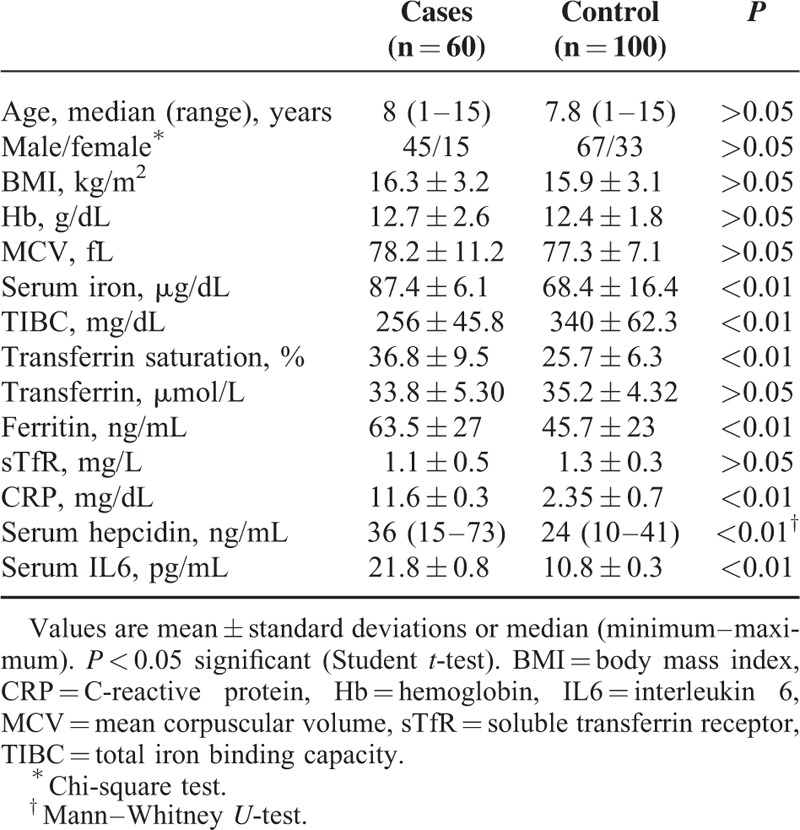
Baseline Clinical and Laboratory Data of the Studied Patients and Control Groups

**FIGURE 1:**
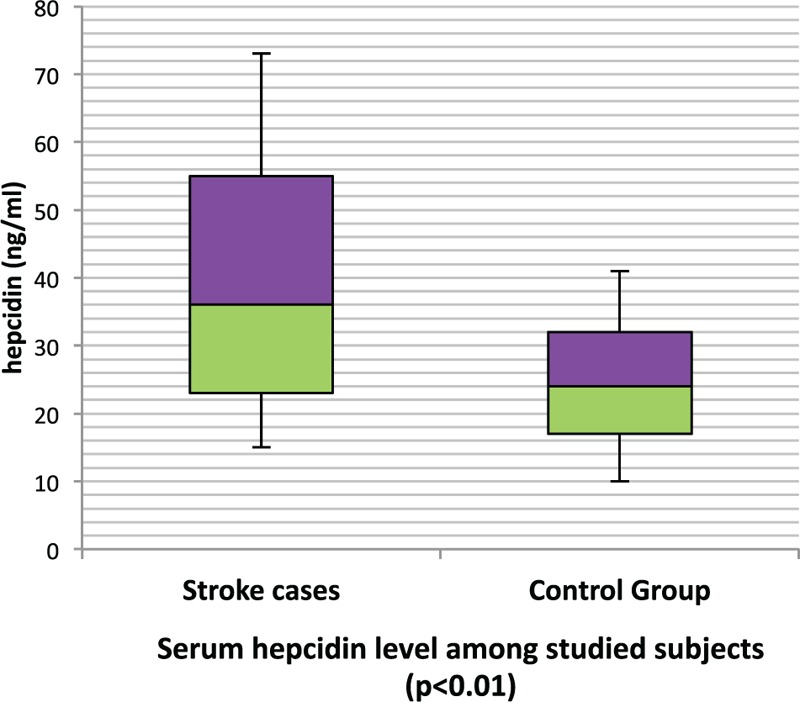
Serum hepcidin level among studied subjects.

Of note, AIS cases showed significantly higher median serum hepcidin level compared to the control group (median, 36 vs 24 ng/mL; *P* < 0.01, respectively) (Figure 1).

On the 1st day of AIS diagnosis, serum hepcidin levels were similar in both stroke subgroups (*P* > 0.05). However, on the 7th day of diagnosis serum hepcidin level decreased significantly in (group 1) cases (median, 36 vs 21 ng/mL; *P* < 0.01, respectively). Meanwhile, no significant change was observed in serum hepcidin level in (group 2) cases (*P* > 0.05); although it persisted at a significantly higher level than group1 (*P* < 0.05), Table 3.

**TABLE 3:**
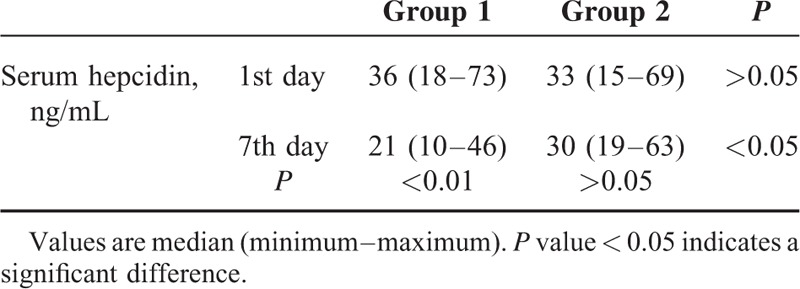
Serum Hepcidin Levels at the Time of Diagnosis (1st day) and 1 week After Diagnosis (7th day) in Patients With Acute Ischemic Stroke (AIS)

Simple correlation analyses were performed to investigate the association of serum hepcidin level with estimated iron parameters and serum IL6in patients with AIS. Serum hepcidin showed significant positive correlations with serum iron, transferrin saturation, ferritin, and IL6 (*r* = 0.375, *P* < 0.05; *r* = 0.453, *P* < 0.05; *r* = 0.687, *P* < 0.01; *r* = 0.515, *P* < 0.01; respectively), Table 4. On the other hand, serum hepcidin showed significant negative correlations with TIBC, transferrin, and sTfR (*r* = −0.526, *P* < 0.01; *r* = −0.377, *P* < 0.05; *r* = −0.539, *P* < 0.01; respectively) Table 4.

**TABLE 4:**
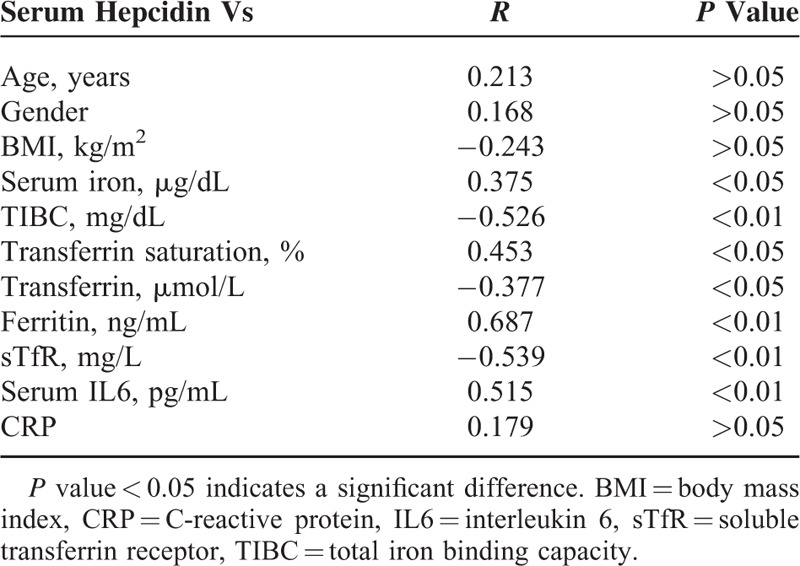
Correlation Between Serum Hepcidin and Clinico-Laboratory Parameters in Patients With Acute Ischemic Stroke

## DISCUSSION

Recently, there has been a considerable interest in the association of iron homeostasis and stroke. Few clinical studies have concluded that iron deficiency anemia may be a more potent risk factor for childhood stroke than previously has been appreciated.^14–16^ However, the factors regulating the expression and activity of iron transport proteins in the brain remain to be identified.

In our study male represented 75% among stroke cases. Interestingly, just as gender differences are found in adult stroke studies, pediatric studies have shown a male predominance in children with a stroke.^16–18^ Among boys, elevated testosterone level was independently associated with stroke risk.^19^ The mechanism of this association is still unknown.

At the beginning of our study, we found that AIS cases had higher serum iron, and ferritin levels compared to the control group (*P* < 0.01). This agreed with a study performed by Chi et al^20^ who reported increased free iron and ferritin levels in ischemic brain. Free iron is a major generator of reactive oxygen species that are capable of damaging biological molecules.^21^ Moreover, during hypoxia, free iron appears to accelerate intracellular free radical formation and lipid peroxidation, causing neuronal injury and death.^22^

On the other hand, we did not observe significant differences between studied groups as regards serum transferrin or sTfR (*P* > 0.05). These results are different from those of Tang et al^23^ who reported that increased hypoxia inducible factor 1 expression, as one of the factors activated in early ischemia, promotes the expression of sTfR that leads to an increase in free iron.

Interestingly, we found that AIS cases had significantly higher serum hepcidin levels in comparison to healthy controls (*P* < 0.01). To date, only a few studies in adult stroke have reported increased levels of plasma hepcidin in AIS victims.^24,25^ Słomka et al^24^ reported that the plasma hepcidin, sHJV, and sTfR levels were significantly higher in 31 patients diagnosed with AIS, compared with 20 healthy control subjects. The authors provide evidence that AIS was associated with increased hepcidin levels and stroke treatment may have an influence on hepcidin synthesis.

Petrova et al^25^ found significantly elevated serum hepcidin levels in AIS adult patients compared to the control group indicating that serum iron and hepcidin levels are a part of the etiology of cerebral ischemia.

Hepcidin, an antimicrobial-like peptide hormone, has emerged as the master regulator of iron metabolism. Hepcidin expression is regulated by body iron status, oxygen tension, erythropoietin activity, and inflammatory cytokines.^26^ Ding et al^8^ confirmed that hepcidin mRNA level and hepcidin/prohepcidin protein levels are upregulated in the ischemic brain. Thus, it is possible that via FPN1 degradation hepcidin reduces the neuronal efflux of iron and, therefore, causes brain iron accumulation.

In the present study, serum hepcidin in AIS cases showed significant positive correlation with serum iron, transferrin saturation, and ferritin (all *P* < 0.01). Among studied iron parameters, serum ferritin was the one showing the best positive correlation with hepcidin at each point during the study (*r* = 0.687; *P* < 0.01). On the other hand, serum hepcidin showed significant negative correlation with TIBC, transferrin, and sTfR. These results are concordant with those of previous reports^27,28^ suggesting that iron itself, or the kinetics of iron use in response to hypoxia, may signal the expression of hepcidin.

Our study confirms the significantly higher IL6 level in AIS cases compared to the controls (*P* < 0.01). Furthermore, we observed a significant positive correlation between serum hepcidin and IL6 levels in AIS cases (*r* = 0.515; *P* < 0.01).

The molecular mechanisms underlying hepcidin regulation by inflammation and ischemia are areas of intense investigation but are still incompletely understood. It is well established that the inflammatory cytokine IL-6 is a major mediator of the inflammatory response and that inflammation upregulates the level of hepcidin. Recent studies^29,30^ pointed out that inflammation fulfills an exceptional role in the pathophysiology of ischemic stroke and ischemia increases IL-6 concentrations in brain tissues.^8^ Briefly, IL-6 can activate the JAK/STAT signaling pathway that upregulates the level of signal transducer and activator of transcription 3. Signal transducer and activator of transcription 3 binds to the HAMP promoter and thus increases hepcidin expression.^31^

On the 1st day of AIS diagnosis, we observed that serum hepcidin levels were similar in both stroke subgroups (*P* > 0.05). However, serum hepcidin decreased significantly on the 7th day of AIS diagnosis in cases treated with (LMWH; enoxaparin sodium) (group1); (median, 36 vs 21 ng/mL; *P* < 0.01, respectively). On the other hand, no significant change was observed in serum hepcidin level in (group 2) cases (*P* > 0.05); although it persisted at a significantly higher level than group 1 (*P* < 0.05). This was concordant with a study performed by Słomka et al,^24^ who confirmed decreased levels of plasma hepcidin in adult AIS victims who received a once-daily or a twice daily injection with the usual pharmacologic doses of LMWH enoxaparin sodium for 4–5 days. Poli et al^32^ discovered that heparin can block hepcidin production in the liver both in vitro and in vivo. The authors also show that heparin abolishes hepcidin induction caused by the inflammatory cytokines IL-6 and Oncostatin M. The majority of the antihepcidin activity of heparin was attributed to its interference with the bone morphogenetic protein-sons of mothers against decapentaplegic (SMAD) signaling pathway, which is a fundamental regulatory mechanism controlling hepcidin synthesis. This is consistent with the bone morphogenetic protein-SMAD-dependent nature of IL-6-mediated induction of hepcidin expression.^33^

To the best of our knowledge, this was the 1st study that shows elevated serum hepcidin level in pediatric AIS. However, the small sample size was one of our limitations in this study; we suggest that multicenter approaches may be necessary to attain larger sample size. Another limitation in our study was unmatched cases in AIS subgroups, as contraindications to LMWH therapy which were present in group II AIS, were absent in group I cases.

Future more extended studies are recommended to confirm our findings and to evaluate the potential effects of LMWH on serum hepcidin level in pediatric AIS patients.

## CONCLUSIONS

Our data brought a novel observation of elevated serum hepcidin level in pediatric AIS patients and pointed out that treatment with LMWH could modulate hepcidin level in those patients.

Finally, a detailed knowledge of the signaling pathway that modulates hepcidin expression should inform a new pharmacologic approach to modifying brain iron homeostasis, offering new treatment modalities of pediatric AIS.
